# Liposome encapsulated Disulfiram inhibits NFκB pathway and targets breast cancer stem cells *in vitro* and *in vivo*

**DOI:** 10.18632/oncotarget.2166

**Published:** 2014-07-04

**Authors:** Peng Liu, Zhipeng Wang, Sarah Brown, Vinodh Kannappan, Patricia Erebi Tawari, Wenguo Jiang, Juan M. Irache, James Z. Tang, Stephen Britland, Angel L. Armesilla, John L. Darling, Xing Tang, Weiguang Wang

**Affiliations:** ^1^ Research Institute in Healthcare Science, Faculty of Science & Engineering, University of Wolverhampton, Wolverhampton, UK; ^2^ Cardiff University-Peking University Cancer Institute, Cardiff University School of Medicine, Henry Wellcome Building, Heath Park, Cardiff, UK; ^3^ School of Pharmacy, University of Navarra, Pamplona, Spain; ^4^ School of Pharmacy, Shenyang Pharmaceutical University, Shenyang, China

**Keywords:** Breast cancer, cancer stem cells, Disulfiram, NFkappaB, chemoresistance

## Abstract

Breast cancer stem cells (BCSCs) are pan-resistant to different anticancer agents and responsible for cancer relapse. Disulfiram (DS), an antialcoholism drug, targets CSCs and reverses pan-chemoresistance. The anticancer application of DS is limited by its very short half-life in the bloodstream. This prompted us to develop a liposome-encapsulated DS (Lipo-DS) and examine its anticancer effect and mechanisms *in vitro* and *in vivo*.

The relationship between hypoxia and CSCs was examined by *in vitro* comparison of BC cells cultured in spheroid and hypoxic conditions. To determine the importance of NFκB activation in bridging hypoxia and CSC-related pan-resistance, the CSC characters and drug sensitivity in BC cell lines were observed in NFκB p65 transfected cell lines. The effect of Lipo-DS on the NFκB pathway, CSCs and chemosensitivity was investigated *in vitro* and *in vivo*.

The spheroid cultured BC cells manifested CSC characteristics and pan-resistance to anticancer drugs. This was related to the hypoxic condition in the spheres. Hypoxia induced activation of NFκB and chemoresistance. Transfection of BC cells with NFκB p65 also induced CSC characters and pan-resistance. Lipo-DS blocked NFκB activation and specifically targeted CSCs *in vitro*. Lipo-DS also targeted the CSC population *in vivo* and showed very strong anticancer efficacy. Mice tolerated the treatment very well and no significant *in vivo* nonspecific toxicity was observed.

Hypoxia induced NFκB activation is responsible for stemness and chemoresistance in BCSCs. Lipo-DS targets NFκB pathway and CSCs. Further study may translate DS into cancer therapeutics.

## INTRODUCTION

Chemoresistance is the major hindrance for the success of advanced/metastatic BC (A/MBC) treatment. The relapsed A/MBC is commonly pan-resistant to a wide range of anticancer drugs, which cannot be explained by individual genetic and biochemical resistant pathway. BC is a highly heterogeneous disease containing a very small fraction (1%) of cancer stem cell (CSC) [1] population with stem cell characters e.g. self-renewal, development into the original tumors in immune deficient mice, differentiation into several linages of progenies. BCSCs can be detected by stem cell markers (ALDH^+^, CD24^Low^/CD44^High^) and activation of embryonic related pathways (Sox2, Oct4, Nanog) [2]. BCSCs are relatively quiescent and pan-chemoresistant [3, 4]. Conventional anticancer drugs fail to eradicate the BCSCs, which become the source of tumor recurrence.

Emerging evidence suggests that CSCs reside in a hypoxic/necrotic tumorous area named the CSC niche [5]. A wide range of studies has demonstrated that hypoxia plays pivotal roles in maintenance of the stemness of CSCs. The hypoxia-induced CSC characteristics can be reversed into a differentiated phenotype in normoxic condition and *vice versa*. *In vitro*, the CSC and non-CSC phenotypes are interconversible in spheroid and monolayer culture respectively [6, 7]. Therefore the environmental oxygen concentration plays determinant roles in maintaining stemness in CSCs [8]. Although most of the publications emphasize the importance of the hypoxia inducible factors (HIFs) in induction and maintenance of CSC phenotypes, the molecular links between hypoxia and CSCs are still not fully elucidated. More than 20 transcription factors including NFκB are induced by hypoxia [9]. Hypoxia induces NFκB activation in a wide range of cells [6]. NFκB plays important role in cell survival, proliferation, invasion, migration and chemoresistance [10, 11]. Human NFκB is composed of 5 subunits [p50/p105, p52/p100, p65 (RelA), RelB and c-Rel], which form homo- or heterodimer binding to DNA target sites (κB sites) to influence downstream gene expression. The most common dimer is a p50-RelA heterodimer. In most normal cells, NFκB is sequestered in the cytoplasm as an inactive complex through the direct binding to its natural inhibitor, the inhibitor of NFκB (IκB). Upon various stimuli, IκB will be phosphorylated, ubiquitinated and promptly degraded which releases NFκB from NFκB-IB complex. The liberated NFκB dimers are then translocated into the nucleus and trigger a series of molecular reactions. Hypoxic activation of NFκB pathway may be HIF1α-dependent. HIF1α may directly interact with NFκB proteins to promote its DNA binding activity [12-14]. In addition to HIF-mediated activation of NFκB, it is recently demonstrated that hypoxia can directly induce NFκB which in turn regulates HIF pathway. The promoter region of HIF1α contains κB site. The activation of NFκB induced by TNFα and p50/p65 transfection lead to increased levels of HIF1α mRNA and protein. Therefore NF-κB can regulate HIF1α signaling pathway to maintain the basal levels of HIF1α under normoxic condition and further induces it under hypoxia [15, 16]. NFκB also induces HIF2α activation via influence of the interaction between IKKγ and CBP/p300 [17, 18]. Recent studies indicate that NFκB plays a pivotal role in hypoxia-induced CSC phenotypes and is responsible for the chemoresistance in CSCs [19].

It is widely accepted that development of anti-CSC drugs to target CSC determinant pathways will improve chemotherapeutic outcomes. Disulfiram (DS), a commercially available anti-alcoholism drug [20], shows anticancer activity *in vitro* and *in vivo* [21-24]. Our previous studies demonstrate that DS enhances 5-fluorouracil, paclitaxel (PTX) and gemcitabine (dFdC) induced apoptosis in colon, breast and brain cancer cell lines [21, 25-27]. The randomized clinical trial indicates that in combination with chemotherapy, ditiocarb, the derivative of DS, significantly improves the 5-year overall survival of high risk BC patients [28]. The anticancer activity of DS is copper (Cu) dependent [22, 29]. Cu plays a crucial role in redox reactions and triggers the generation of reactive oxygen species (ROS) in human cells. DS/Cu is a strong ROS inducer [30] and proteasome-NFκB pathway inhibitor [21, 22, 25]. DS specifically inhibits the activity of aldehyde dehydrogenase (ALDH), a functional marker of CSCs and ROS scavenger [31, 32]. Combination of DS with Cu may target cancer cells by simultaneous modulation of both ROS and NFκB. DS and its metabolites can also permanently inhibit Pgp activity [33].

Although the anticancer activity of DS has been reported for a long time, only very few successful cases have been reported in clinic [28, 34]. This discrepancy may be mainly introduced by the very short half-life of DS in the bloodstream. Nano-technology may be able to extend the half-life of DS and translate it into cancer indication. In this study, we investigated the effect of hypoxia on CSCs and elucidated the bridging role of NFκB in linking hypoxia and CSCs. We also examined the *in vitro* and *in vivo* anticancer efficacy of a newly developed liposome-encapsulated DS (Lipo-DS). Our data indicate that NFκB plays a key role in pan-resistance of hypoxia-induced CSCs. Lipo-DS can efficiently abolish CSCs and reverse chemoresistance.

## RESULTS

### Hypoxia is responsible for maintaining stemness and drug resistance in mammosphere (MSC) and suspension cells (SUS)

**Figure 1:**
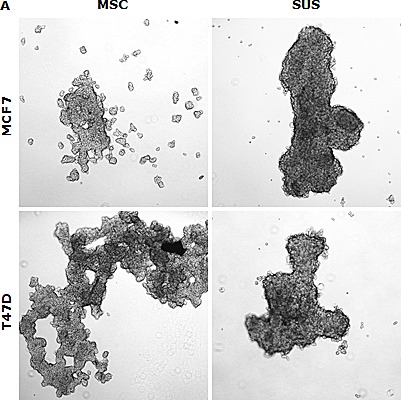

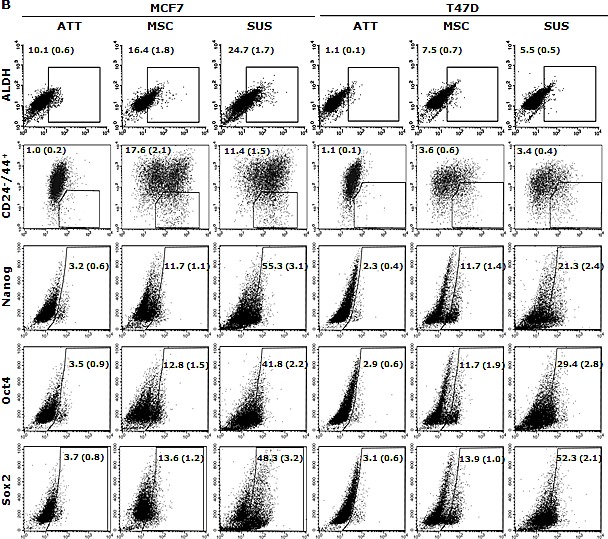

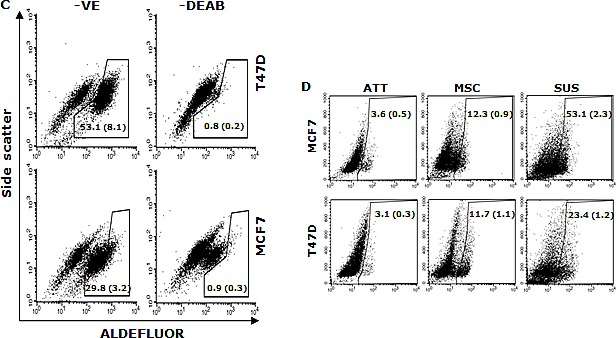

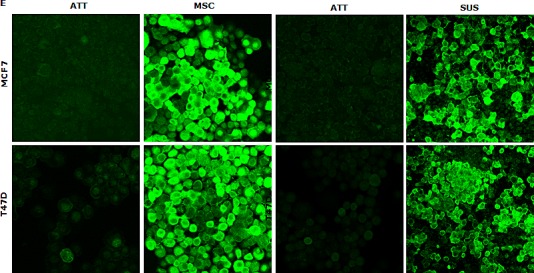

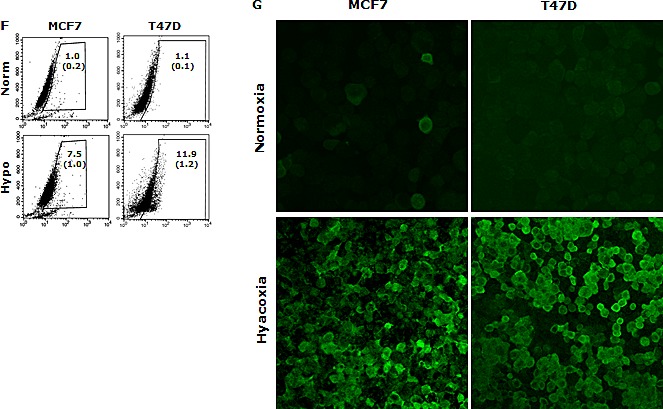

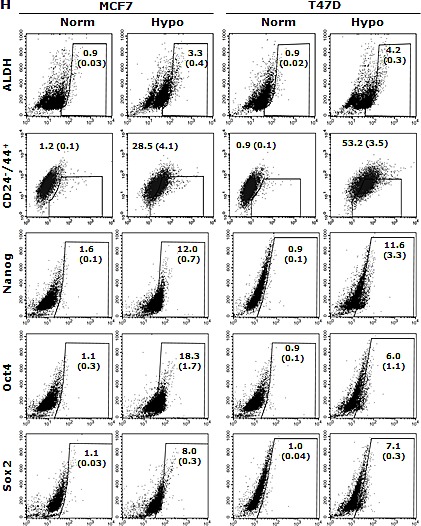
Hypoxia induces expression of stem cell markers in BC cell lines A. The morphology of spheres cultured in serum-free (MSC) and serum-containing (SUS) medium (40× magnification). B. Flow cytometric analysis of ALDH activity and expression of CD24, CD44, Oct4, Sox2 and Nanog proteins in monolayer- and suspension-cultured cells. In comparison with the attached cells, all of these markers are expressed significantly higher in MSC and SUS (p<0.01). C. Specificity of ALDEFUOR in detection of ALDH activity in BC cell lines. DEAB: specific inhibitor of ALDH. D. Flow cytometric detection of hypoxic cells stained with Hypoxyprobe. Significantly higher population of hypoxic cells was detected in MSC and SUS cells (p<0.01). E. Confocal microscopy images of the hypoxic cells detected by Hypoxyprobe in serum-free (MSC) and serum-containing (SUS) medium cultured BC cells (×400 magnification). F and G. Hypoxyprobe stained hypoxic population in hypoxia-cultured BC cells was detected by flow cytometry (F) and immunocytochemistry (G) respectively. H. Flow cytometric comparison of ALDH activity and expression levels of CD24, CD44, Oct4, Sox2 and Nanog proteins in normoxia- and hypoxia-cultured cells. In comparison with the normoxic cells, stem cell markers were detected in significantly higher population of the hypoxic cells (p<0.01). ATT: monolayer culture; MSC: serum-free stem cell culture; SUS: serum-containing medium culture; Norm: normoxia; Hypo: hypoxia. The numbers in the frame represent Mean (SD) from three experiments.

In this study, we examined if the traditional stem cell culture system is essential for maintaining the stemness *in vitro*. Two breast cancer cell lines were cultured in both classical serum-free spheroid stem cell culture system and serum-rich (10%) medium in parallel. After 7 days culture, BC cells formed typical mammospheres in both conditions. The serum-free medium cultured cells formed numerous relatively smaller spheres which loosely aggregated together. In contrast, the cells cultured in the serum-rich medium formed markedly larger and tighter spheres (Fig. 1A). Furthermore, we compared the expression of stem cell markers and CSC-related embryonic proteins in these cells. Both MSCs and SUS cells have significantly higher proportion of cells expressing stem cell markers (ALDH^+^ and CD24^low^/CD44^high^) and CSC-related embryonic proteins (Sox2, Nanog and Oct4). In comparison with the MSCs, most of these proteins are expressed at higher levels in the SUS cells (Fig. 1B). CSCs commonly possess *de novo* resistance to a wide range of anticancer drugs [1]. Furthermore we examined the chemosensitivity in these cells. Table 1 shows that resistance of BC cells to three first line anti-BC drugs was induced in both culture systems. These results suggest that the stemness and chemosensitivity in BC cells were not governed by the components in the culture medium. It has been reported that the hypoxic condition in the stem cell niche is essential for maintaining the stemness and chemoresistance [6]. We hypothesized that the hypoxic condition in the mammospheres may play the role in maintenance of stemness and chemoresistance. Fig. 1D and 1E demonstrate that in comparison with the adherent cells, high population of hypoxic cells were detected in both MSC and SUS cells by HypoxyProbe. Furthermore we cultured both cell lines in hypoxic condition (1% O_2_) for 5 days to determine the relationship between hypoxia and MSC characteristics. Fig. 1F to 1H show that the hypoxia-cultured monolayer cells express MSC markers and embryonic proteins. Similar to the MSC and SUS cells, the cells cultured in hypoxic condition are significantly resistant to chemotherapeutic agents (Table 1). All of these data indicate that hypoxia may play a key role in determination of stemness and chemosentivity in BC cells.

**Table 1:** Cytotoxicity of conventional anticancer drugs in BC cell lines

	MCF7			T47D		
	dFdC	Dox	PTX	dFdC	Dox	PTX
ATT	28.7 (7.6)	17.1 (1.1)	6.3 (1.6)	76.3 (5.7)	64.2 (2.4)	7.7 (2.4)
MSC	>1000	>2000	>1000	>1000	>2000	>1000
SUS	>1000	>2000	>1000	>1000	>2000	>1000
Hypo	>1000	>2000	>1000	>1000	>2000	>1000
Mock	14.8 (5.2)	63.2 (12.9)	2.8 (0.4)	2.5 (2.0)	169.8 (42.5)	6.2 (1.8)
C2	>1000	424.3** (65.5)	>1000	N/A	N/A	N/A
P1	>1000	159.8** (36.8)	>1000	N/A	N/A	N/A
C1	N/A	N/A	N/A	>1000	498** (130)	>1000
C3	N/A	N/A	N/A	>1000	>1000	>1000

The figure represents IC_50_ value from three MTT experiments [mean (SD)]. The cells were exposed to drugs for 72 hours. ATT: attached cells; MSC: mammosphere cells; SUS: suspension culture in normal medium; Hypo: hypoxic culture (O_2_ < 1%). Mock: Empty vector transfected cells; C2, P1, C1, C3: NFκB p65 transfected clones. ** p<0.01 (n=3) compared with Mock cells.

### NFκB activation plays a pivotal role in maintaining CSC stemness and chemoresistance

**Figure 2:**
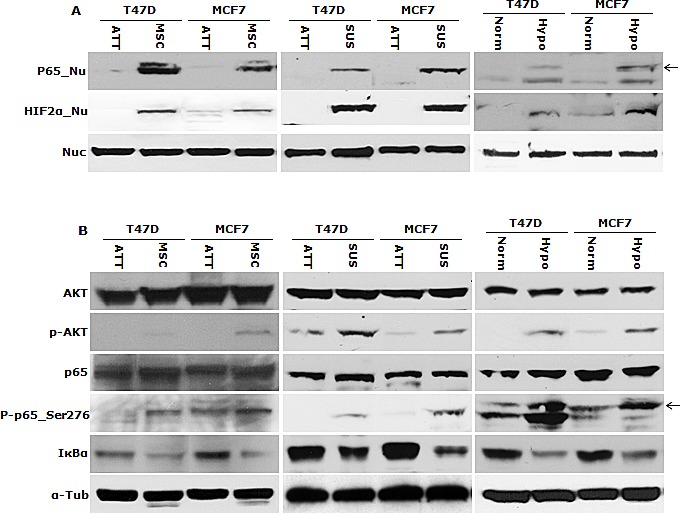

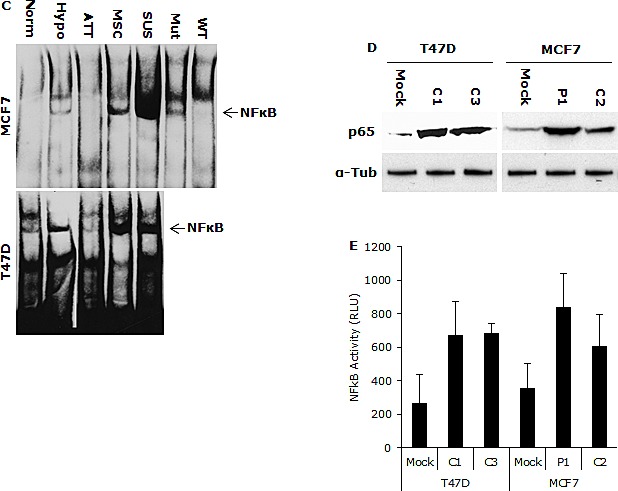

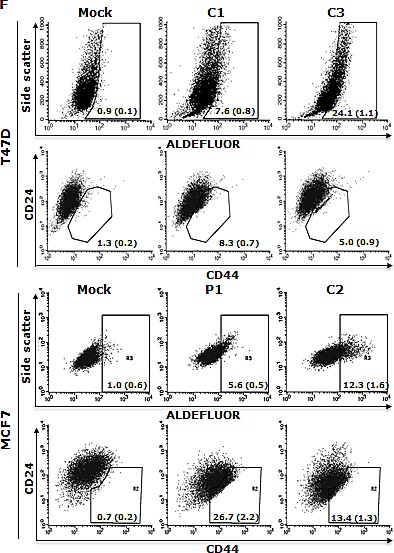
NFκB is responsible for maintaining CSC characters in suspension- and hypoxia-cultured cells A. High levels of NFκB p65 and HIF2α protein were detected in the nuclear protein extracted from suspension- and hypoxia-cultured cells. Nuc: nucleolin used as a loading standard. B. Phosphorylated AKT, NFκB p65_Ser276 and degradation of IκBα were detected in suspension- and hypoxia-cultured cells by western blot. α-Tub: α-tubulin used as a loading control. C. High NFκB DNA binding activity was detected by EMSA. Mut and WT: mutant and wild type probe competition. D and E. High NFκB p65 protein (D) and transcriptional activity (E) were detected in p65 transfected clones (C1, C3, P1 and C2) by western blot and luciferase reporter gene assay respectively. Mock: empty vector transfected cells. F. High ALDH^+^ and CD24^low^/CD44^high^ population was detected in NFκB p65 transfected clones.

High HIF2α nuclear protein was detected in the cells cultured in hypoxia, MSC and SUS conditions. IκBα degradation and NFκB p65 nuclear translocation were detected (Fig. 2A and 2B). NFκB p65 and AKT Phosphorylation and increased NFκB DNA binding activity were also detected in these cells (Fig. 2B and 2C). These results indicate that NFκB may be the pivotal factor in hypoxia-induced CSC characteristics. To examine the importance of NFκB in maintenance of stemness and chemosensitivity, both MCF7 and T47D cell lines were transfected with NFκB p65 subunit. High p65 protein levels and transcriptional activity of NFκB were detected in the transformed clones (Fig. 2D and 2E). Fig. 2F shows that the transformed clones possess significantly higher (p<0.01) population of CSCs (ALDH^+^, CD24^low^/CD44^high^). In comparison with the mock-transfected cells, the p65 transfected cell lines are highly resistant to three first line anti-BC drugs [doxorubicin (Dox), PTX and dFdC] (Table 1). These data suggest that NFκB plays a key role in hypoxia-induced CSCs and chemoresistance.

### Lipo-DS targets CSCs *in vitro*

**Figure 3:**
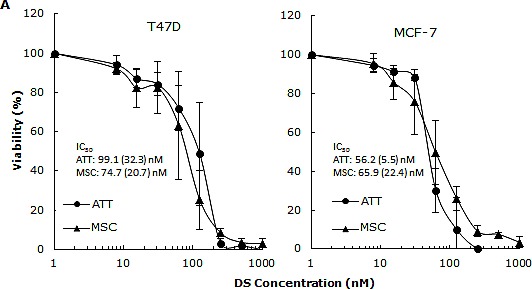

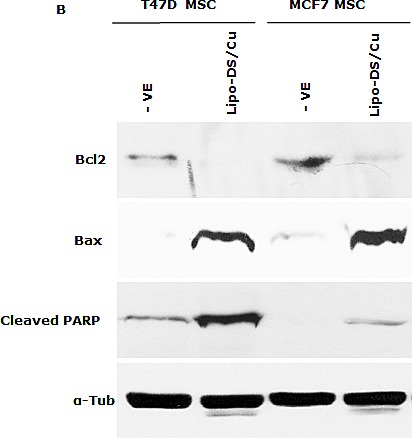

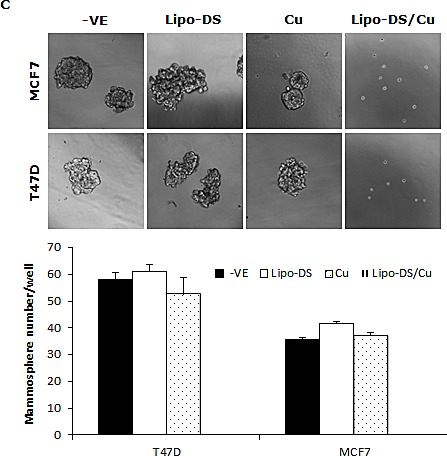

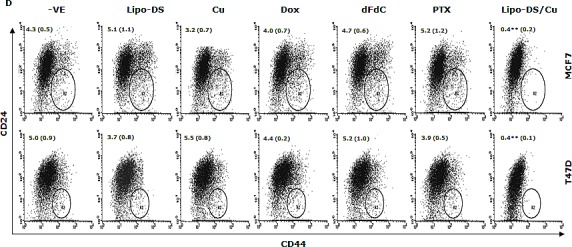

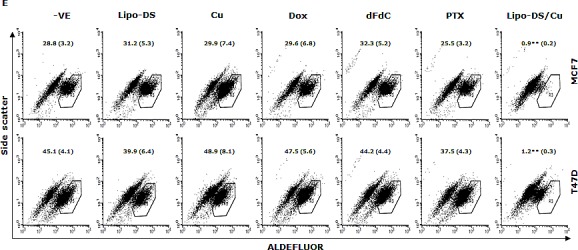

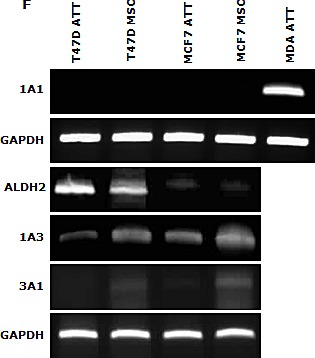

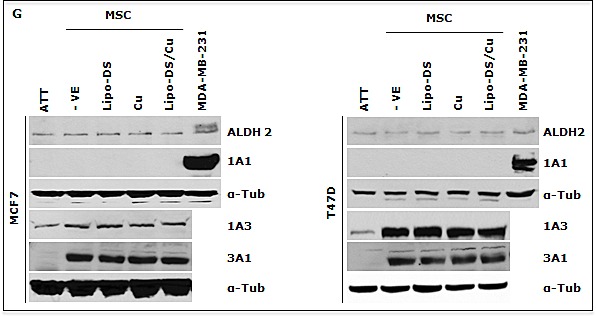
Cytotoxic effect of Lipo-DS/Cu on BCSCs A. Comparable cytotoxicity of Lipo-DS/Cu in monolayer-cultured cells and BCSCs was detected by MTT assay. The cells were exposed to Lipo-DS/Cu for 72 hours. B. Lipo-DS/Cu induced Bax and cleaved PARP and reduced Bcl2 expression were detected by western blot. The cells were treated in Lipo-DS (1μM)/CuCl_2_(10μM) for 4 hours and in drug-free free stem cell culture medium for 24 hours. C. Lipo-DS/Cu abolished sphere-forming ability in BC cell lines. The cells were cultured in Lipo-DS (1μM), CuCl_2_ (10μM) or Lipo-DS/CuCl_2_ for 4 hours and in drug-free free stem cell culture medium for 7 days. D. The effect of different treatments on CD24^low^/CD44^high^ population. E. The effect of different treatments on ALDH activity in BCSCs. For experiments D and E, 7-day-cultured sphere cells were trypsinized and exposed to different agents for 4 hours, then released for 24 hours. (** in D and E: In comparison with other groups, p<0.01) F. Expression of ALDH mRNAs in attached and spheroid cells. G. Lipo-DS/Cu did not influence the expression of ALDH proteins in CSCs. The whole proteins were extracted from the BCSCs exposed to different agents for 4 hours and released in drug-free medium for 24 hours.

CuCl_2_ (10μM) was used in all the experiments because Cu is essential for DS induced cytotoxicity [21, 27, 35]. Lipo-DS/Cu was highly cytotoxic to MSCs (Fig. 3A) although these cells are resistant to conventional anti-BC drugs (Table 1). Lipo-DS/Cu induced and inhibited expression of Bax and Bcl2 proteins respectively (Fig. 3B). The sphere-forming ability was also completely abolished after 4 hours exposure to Lipo-DS/Cu but not Lipo-DS and Cu alone (Fig. 3C). The ALDH^+^ and CD24^low^/CD44^high^ CSC population in the mammospheres was eliminated by Lipo-DS/Cu but not Lipo-DS, Cu and anti-BC drugs (Fig. 3D and 3E). Four ALDH isoenzymes (1A1, 1A3, 2 and 3A1) are most possible to be involved in the ALDH activity in CSCs [32, 36-38]. The mRNA and protein of these isoenzymes were examined to determine if Lipo-DS also inhibits ALDH at transcriptional and translational levels. Although most of the previous publications mentioned that ALDH1A1 is responsible for ALDH activity in CSCs, we could not detect its expression in both attached and sphere-cultured MCF7 and T47D cells. ALDH2, a mitochondria-located isoenzyme, was expressed at background levels and not induced in CSCs. In contrast, the mRNA and protein of ALDH1A3 and 3A1 were expressed at very low levels in the attached cells and markedly induced by MSC culture (Fig. 3F and 3G). Therefore ALDH1A3 and 3A1 may contribute to the high ALDH activity in the CSCs derived from MCF7 and T47D cell lines. Although Lipo-DS/Cu inhibited ALDH activity but it had no effect on the mRNA and protein expression of these 4 isoenzymes.

### Lipo-DS/Cu simultaneously triggers ROS-MAPK activation and inhibits NFκB pathway in MSCs

**Figure 4:**
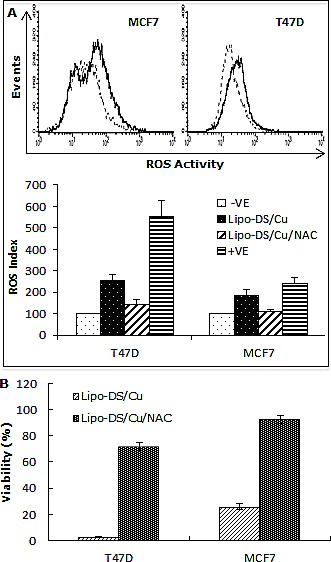

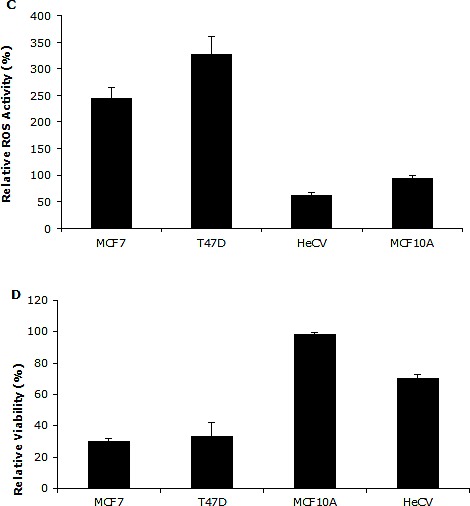

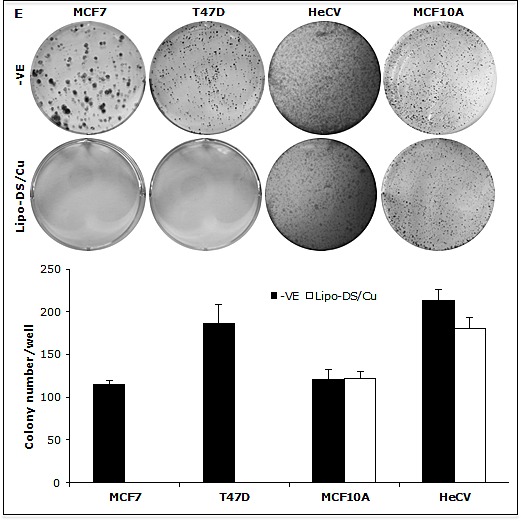

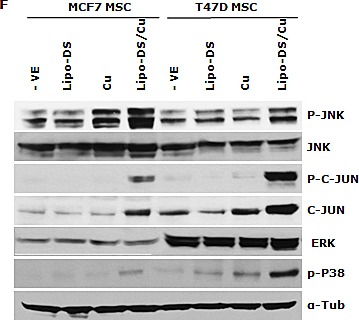

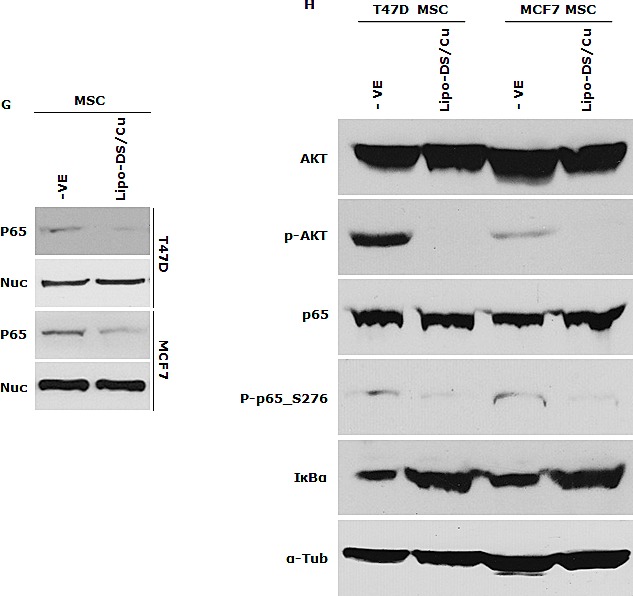
Lipo-DS/Cu induces ROS-MAPK and inhibits NFκB pathways and specifically targets BC cells A. Top: Flow cytometric detection of Lipo-DS/Cu-induced ROS activity in BC cells. Dot-line: control, Solid-line: Lipo-DS/Cu. Bottom: Lipo-DS/Cu-induced ROS activity was reversed by NAC. +VE: positive control (Pyocyanin 200μM). B. The Lipo-DS/Cu-induced cytotoxicity was reversed by NAC. C. Lipo-DS/Cu specifically induced ROS activity in BC cell lines. Relative ROS activity = (Treated/untreated) × 100%. D. Lipo-DS/Cu demonstrated specific cytotoxicity in BC cell lines. Relative viability = (Treated/untreated) × 100%. E. Clonogenic assay shows that Lipo-DS/Cu abolished colony-forming ability in BC cell lines but no effect on normal cell lines. The cells were exposed to drugs for 1 hour and released in drug free medium for 72 hours (MTT) and 10 days (clonogenic assay). F. The effect of different treatments on JNK, p38 and ERK pathways. G. Lipo-DS/Cu blocked NFκB p65 nuclear translocation. H. Lipo-DS/Cu induced IκBα expression and inhibited phosphorylation of AKT and NFκB p65. For F – H, 6-day-cultured spheres were trypsinized and exposed to different treatments (Lipo-DS 1μM, Cu 10μM, Lipo-DS/Cu) for 4 hours and release in drug-free medium for 24 hours.

We previously reported that DS/Cu activates ROS-MAPK and inhibits NFκB pathways in attached cells [21]. In this study, we examined the effect of Lipo-DS/Cu on MSCs. Lipo-DS/Cu induced ROS activity in MSCs was reversed by NAC, a ROS inhibitor. NAC also reversed Lipo-DS/Cu induced cytotoxicity (Fig. 4A and B). In comparison with normal cell lines, Lipo-DS/Cu selectively induced higher ROS activity and cytotoxicity in cancer cells (Fig. 4C and 4D). After exposure to Lipo-DS/Cu for one hour, the clonogenicity in cancer cell lines was completely abolished but no significant effect was observed in Lipo-DS/Cu treated normal cells (MCF10A and HeCV)(Fig. 4E). After exposure to Lipo-DS/Cu for 4 hours, the major MAPK pathway elements, e.g. phosphorylated JNK, phosphorylated C-JUN and phosphorylated p38 but not ERK, were significantly induced (Fig. 4F). In contrast to the MAPK pathway, Lipo-DS/Cu inhibited IκBα degradation and blocked NFκB p65 nuclear translocation in both MCF7 and T47D CSCs. The phosphorylation of NFκB p65 and AKT in the MSCs was also inhibited by Lipo-DS/Cu (Fig. 4G and 4H).

### Disulfiram inhibits BC xenografts *in vivo*

**Figure 5:**
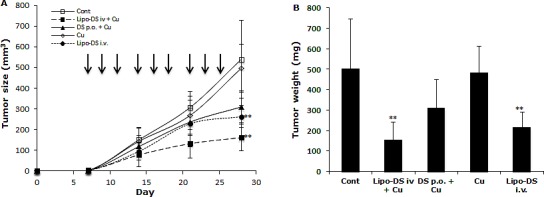

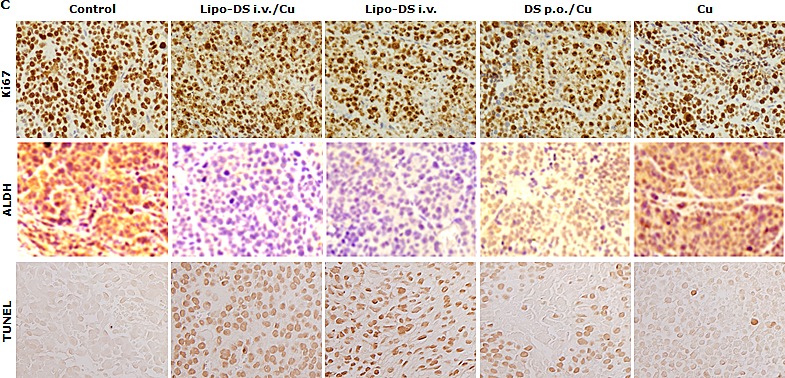

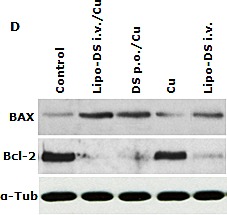

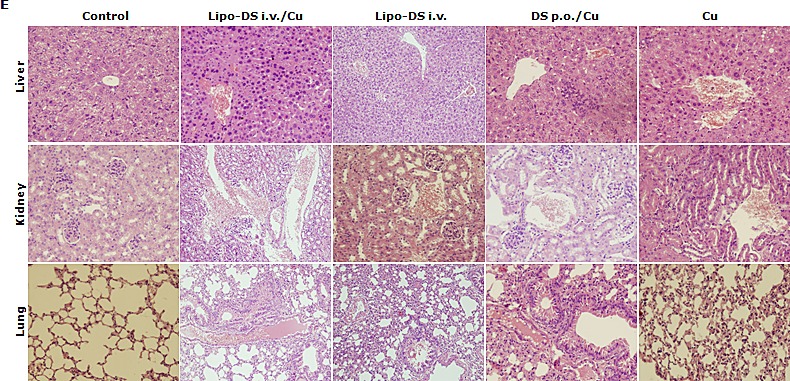
Lipo-DS inhibited growth of BC xenografts MDA-MB-231 cells (1×10^6^) were subcutaneously injected at one rear flank of the mice. When the tumor volume reached ~200mm^3^, the tumor bearing mice were randomly subdivided into 5 groups (8 mice/group) e.g. control; DS 75mg/kg p.o + CuGlu 8mg/kg p.o; CuGlu 8mg/kg p.o; Lipo-DS 75mg/kg i.v; Lipo-DS 75mg/kg i.v + CuGlu 8mg/kg p.o. The drugs were administered 3 times/week for successive 3 weeks. A. Growth curves of tumor size. Arrows represent drug administration. B. The effect of different treatments on tumor weight after 28 days observation. C. The effect of different treatment on Ki67 and TUNEL expression (×400 magnification). D. The effect of different treatments on BAX and Bcl-2 expression. E. Histo-pathological images of vital organs (H&E staining, 400 magnification).

Furthermore, the anticancer efficacy of Lipo-DS was examined *in vivo*. The drug was administered 3 times/week for successive 3 weeks. To determine the necessity of copper supplement *in vivo*, the Lipo-DS i.v with/without copper supplement and copper alone were compared. The oral administration of DS/copper gluconate (CuGlu) was compared with i.v administration of Lipo-DS plus oral administration of CuGlu. In comparison with the control group, both Lipo-DS/CuGlu and Lipo-DS significantly inhibited tumor growth but Lipo-DS/CuGlu demonstrated highest anticancer efficacy (Fig. 5A and 5B). Oral administration of DS/CuGlu also showed tumor inhibiting effect although it is not statistically significant. Copper alone had no effect on tumor growth. Lipo-DS/CuGlu, Lipo-DS and DS/CuGlu induced BAX, inhibited Bcl-2 and induced TUNEL staining (Fig. 5C and 5D). In comparison with the control group, the ALDH expression in tumor tissues was inhibited when the animal were treated with Lipo-DS/CuGlu and Lipo-DS (Fig. 5C). It seems that Lipo-DS/CuGlu had no effect on proliferation because the expression of Ki67 was not affected by the treatments. Administration of CuGlu with or without DS or Lipo-DS induced moderate dilation of blood vessels in the examined organs. No cytotoxic effect was observed in lung and kidney but some necrotic cells were observed in the liver of Lipo-DS/CuGlu treated animals (Fig. 5E).

## DISCUSSION

Since Al-Hajj identified CSCs in BC [39], the existence of CSCs has been reported in several other cancer types and in developed cancer cell lines [40]. The CSC culture system is adapted from the normal stem cell culture system. The serum-free and growth factors-supplemented spheroid culture has been widely used as a gold method *in vitro* to prevent differentiation, purge the differentiated progenies and enrich stem cell population [41]. In contrast to the relatively nonreversible differentiation in normal stem cells, the monolayer-cultured non-CSCs and sphere-cultured CSCs are completely reversible [6, 41]. In comparison with the stem cell culture system, the serum-rich suspended culture system is less costly and has better physiological relevance. In this study, we compared the BC cells cultured in traditional serum-free stem cell medium and serum-rich (10% FCS) medium to determine the necessity of the stem cell medium in maintaining the CSC status. After 6 days culture, the BC cells in both systems formed spheres and clusters (Fig. 1A). It is widely accepted that if it is not a single cell inoculation, the aggregation of the suspended cells is inevitable, no matter how low the cell density ([41] and our unpublished data). Therefore, these spheres and clusters represent both cell proliferation and aggregation. The expression of stem cell and embryonic markers was significantly induced in the cells cultured in both systems. This result indicates that the stemness was not determined by the components in the culture medium. If so, there must be some common feature(s) in the culturing systems which introduce and maintain stemness. Hypoxia is a determinant of stemness in stem cell niche [6]. We hypothesized that due to the penetrating distance hypoxia may be cumulated within the spheres. The cells located in the center of the spheres will show CSCs’ characteristics. Compared with the monolayer-cultured cells, a high population of hypoxic cells was detected in the sphere-cultured cells (Fig. 1D and E). More hypoxic cells were detected in serum-containing spheres. This may be due to that these spheres are significantly bigger and tighter than those in serum-free culture (Fig. 1A). A high population of CSCs was also detected in hypoxia-cultured (<1% O_2_) BC cells. The BC cells cultured in these three conditions are highly resistant to dFdC, Dox and PTX. Chemoresistance is another common feature of CSCs. These results indicate that the stem cell characters in the sphere-cultured cells may be introduced by hypoxia. The serum-free and growth factors-supplemented medium is not essential for the *in vitro* CSC culture.

Although hypoxia can induce stem cell characters, the detailed molecular mechanisms and pathways linking hypoxia and CSCs are still largely unknown. It is generally believed that hypoxia-activated HIF pathways are important for cellular response to hypoxia [6]. However, many other transcription factors, e.g. NFκB, are also crucial for hypoxia-induced phenotypes. Activation of NFκB pathway enables the survival of the inflammatory cells in inflammation-induced hypoxia [6]. Recent studies indicate that NFκB also plays a pivotal role in CSCs and chemoresistance [42-44]. In our study, nuclear translocation of HIF2α and NFκB p65 protein was detected in sphere- and hypoxia-cultured cells. These cells also demonstrated IκBα degradation and p65 phosphorylation. The activation of AKT, the transcription factor upstream of NFκB, was also observed. High NFκB DNA binding activity was induced in all three conditions. Therefore NFκB pathway is activated in all of the three culture systems (MSC, SUS and hypoxia). In order to examine the importance of NFκB in determination of CSC traits, we transfected BC cells with NFκB p65. The transfected clones expressed high levels of BCSC markers (Fig. 2F) and were highly resistant to dFdC, Dox and PTX (Table 1). Therefore NFκB definitely conferred CSC traits upon BC cells.

DS is an NFκB inhibitor which targets CSCs and reverses chemoresistance [21, 25, 27, 35, 45]. The application of DS in cancer therapeutics is limited by its very short half-life in the bloodstream [46]. To overcome this hindrance, we recently developed a Lipo-DS to extend the half-life of DS in the bloodstream [47]. In line with our previous reports [21, 27], the cytotoxicity of Lipo-DS is Cu dependent. After very short exposure (4 hours) to Lipo-DS/Cu, the expression of BCSC markers (ALDH^+^, CD24^low^/CD44^high^) in the sphere cells was inhibited and the sphere-forming ability in the BC cell lines was completely abolished. The BCSC markers were not affected by conventional anticancer drug, Lipo-DS or Cu. Although it was reported that ALDEFLOUR detects enzyme activity of ALDH1A1 [32], we could not detect ALDH1A1 mRNA and protein expression in both cell lines. ALDH2, a mitochondrial isoenzyme, was detected at very low basal levels but not induced by spheroid and hypoxic culture. Marcato et al. [36] reported that ALDH1A3 is responsible for the high ALDH activity in BC. Our study indicates that both ALDH1A3 and 3A1 may be responsible for the high ALDH activity in BCSCs because high and inducible expression of ALDH1A3 and 3A1 mRNA and proteins was detected in sphere-cultured cells. Lipo-DS/Cu had no effect on ALDH isoenzymes (1A1, 1A3, 2 and 3A1) at mRNA and protein levels.

Selectivity is one of the key issues for anticancer drug development. The cytotoxicity of Lipo-DS/Cu is ROS dependent. ROS induces apoptosis by damaging DNA, RNA and protein. Cancer cells possess and also tolerate significantly higher levels of ROS due to the balance of anti-apoptotic mechanisms e.g. NFκB [48]. Lipo-DS/Cu selectively induced ROS activity and showed significantly higher cytotoxicity in BC than normal cell lines. Similar phenomenon was also obtained by clonogenic assay (Fig. 4E). ROS induced-apoptosis is highly MAPK pathway-dependent. Lipo-DS/Cu persistently activated JNK, C-Jun and p38 MAPK pathways but had no effect on ERK pathway which is responsible for cell growth, proliferation and survival [49]. ROS also trigger the expression of anti-apoptotic proteins which in turn neutralize the pro-apoptotic effects of ROS [48]. NFκB is one of the most important ROS-induced anti-apoptotic factors [48]. NFκB inhibits JNK and p38 activation and suppresses ROS accumulation in cancer cells [48, 50]. Cancer cell fate is highly dependent on the crosstalk between JNK/p38 and NFκB pathways. Simultaneous activation of ROS-JNK/p38 and inhibition of NFκB pathway (Fig. 4F – 4H) may contribute to Lipo-DS/Cu induced cytotoxicity in the BC cell lines.

In this study, we first reported the anticancer effect of Lipo-DS in combination with copper *in vivo*. Lipo-DS/CuGlu demonstrated the strongest anticancer efficacy. Lipo-DS injection alone also showed anticancer activity although lower than that of Lipo-DS/CuGlu. This may be due to the intrinsic copper in plasma and tumor tissues [51]. Oral version of DS/Cu showed mild *in vivo* anticancer activity. The Lipo-DS, Lipo-DS/Cu and DS/Cu induced apoptosis was evidenced by TUNEL and western blotting results (Fig. 6C and 6D). Consistence with our *in vitro* data ([21] and unpublished data) Ki67 staining (Fig. 6C) indicates that the anticancer effect of DS and Lipo-DS is proliferation-independent. In line with the *in vitro* data, Lipo-DS inhibited the ALDH^+^ CSC population in the xenografts (Fig. 6C). To determine the *in vivo* selectivity DS, the histo-pathological changes of vital organs (liver, lung and kidney) were examined (Fig. 6E). CuGlu induced blood congestion in major organs. There was no significant toxicity observed in the vital organs except that some necrotic cells were observed in the liver of Lipo-DS/Cu treated mice. Our unpublished data indicate that in combination with Cu, the *in vivo* anticancer effect of nano-encapsulated DS could be achieved at significantly lower dose with no toxicity in liver. Therefore the dose of Lipo-DS in Lipo-DS/CuGlu group still needs to be adjusted. After very short exposure (1 hour) *in vitro*, Lipo-DS showed high selectivity and strong irreversible cytotoxicity in cancer cells. This phenomenon gives us a further indication that development of long-circulating formulation of DS may translate this drug into clinical cancer therapeutics.

## MATERIALS AND METHODS

### Cell lines and reagents

The breast cancer cell lines MCF7, MDA-MB-231, T47D and normal breast epithelial cell line MCF10A were purchased from ATCC (Middlesex, UK). HeCV, the normal human vascular endothelial cell line, [52] was kindly provided by Prof W Jiang (Cardiff University, UK). Gemcitabine (dFdC), doxorubicin (Dox), paclitaxel (PTX), copper(II) chloride (CuCl_2_), copper gluconate (CuGlu), *N*-acetyl-cysteine (NAC) and poly-2-hydroxyethyl methacrylate (poly-HEMA) were purchased from Sigma (Dorset, UK). Lipo-DS was provided by Prof X Tang (Shenyang Pharmaceutical University, China). The drug loading content of Lipo-DS is 3mg/ml. The cumulative release of DS from liposome in 120h is more than 60%. The pharmacokinetic study of DSF in rat plasma after intravenous administration of a dose of 36 mg/kg has been investigated (t_1/2_ =0.1 h and t_1/2d_=0.3 h). The comparison of the biological activity between Lipo-DS and conventional DS has been published [53].

### Cell culture and cytotoxicity analysis

All cell lines were cultured in DMEM (Lonza, Wokingham, UK) supplemented with 10% FCS, 2mM L-glutamine, 50 units/ml penicillin, 50 μg/ml streptomycin. For *in vitro* cytotoxicity assay, the cells (5,000/well) were cultured in 96-well flat-bottomed microtiter plates overnight and exposed to anticancer drugs or Lipo-DS plus CuCl_2_ (10μM) for 72 h, then subjected to a standard MTT assay [54].

### *In vitro* mammosphere and suspension culture and cytotoxicity assay

To culture the BC spheres, cells were cultured in poly-HEMA coated ultra-low adherence flasks or plates to prevent cell adhesion. The spheres were cultured, at a density of 10,000 cells/ml, as MSCs or suspension cells (SUS) in stem cell culture medium [SCM, serum-free DMEM-F12 supplemented with B27(Invitrogen, Paisley, UK), 20 ng/ml epidermal growth factor (EGF, Sigma), 10 ng/ml basic fibroblasts growth factor (b-FGF, R & D System, Abingdon, UK), 10 μg/ml insulin (Sigma)] and full medium (DMEM supplemented with 10% FCS, 2mM L-glutamine, 50 units/ml penicillin, 50 μg/ml streptomycin) respectively. After 6 days culture, the MSC and SUS cell clusters and spheres were photographed and subjected to further treatments. For *in vitro* cytotoxicity assay, the MSC or SUS cultured cells were trypsinised and seeded in 96-well plated at a density of 5,000 cells/well and exposed to drugs for 72 h before MTT assay. CuCl_2_ (10 μM) supplemented culture medium was used in the MTT assay to determine the cytotoxicity of Lipo-DS in MSC and SUS.

### Measurement of hypoxia in cell culture

The hypoxic status was determined using the Hypoxyprobe^TM-1^ Plus Kit supplied by Hypoxyprobe Inc (Burlington, MA, USA) following the supplier's instruction. For immunocytochemistry assay, the cells were cultured in 8-well chamber slides at normoxic (20% oxygen) or hypoxic [1% oxygen in Hypoxic Chamber (StemCell, Durham, NC, USA)] condition for 24 hours and labeled with Hypoxyprobe for 2 hours. The hypoxic cells were detected by confocal microscope after stained with FITC-conjugated anti-hypoxyprobe MAb. The CSCs and cells in suspending condition were cultured for 6 days. The MSCs and suspension-cultured cells were labelled with Hypoxyprobe for 24 hours and cytospined at 800 rpm for 3 min to spread the spheres onto Polylysine-coated slides (VWR, Lutterworth, UK). For flow cytometric analysis, the cells were cultured in 25cm flasks at the above conditions. After immunocytochemistry staining the cells were subjected to flow cytometric analysis. The hypoxic population was detected using a FACSCalibur flow cytometer with 488-nm blue laser and standard FITC 530/30 nm bandpass filter. To determine the effect of hypoxia on drug sensitivity, the cells were cultured in 1% oxygen condition at a cell density of 1×10^3^ cells/well in 96-well plate for 4 days and exposed to anticancer drugs for another 72 h before MTT assay. The parallel MTT assay was performed in normoxic condition.

### Detection of ALDH positive population

The ALDH positive population was detected by ALDEFLUOR kit (StemCell Tech., Durham, NC, USA) following the supplier's instruction. The cells (2.5 × 10^5^) were analyzed after stained in ALDH substrate containing assay buffer for 30 min at 37°C. The negative control was treated with diethylaminobenzaldehyde (DEAB), a specific ALDH inhibitor.

### Flow cytometric analysis of CD24 and CD44 expression

The adherent or mammosphere cells were trypsinised and passed through a 25G needle. The cells (2.5 × 10^5^) were incubated with CD24 and CD44 antibodies (BD Pharmingen, Oxford, UK) for 20 min at 4°C. Unbound antibodies were washed off with 2% FCS HBSS (Sigma) and the cells (10,000 events) were examined no longer than 1 hour after staining on a BD Facscalibur.

### Immunofluorescent flow cytometric analysis of embryonic stem cell markers

The expression of Nanog, Oct4 and Sox2 was determined by immunofluoresent flow cytometry. The sphere and hypoxia-cultured cells were collected by trypsinization. The cells fixed by acetone/methanol and permeabilized by 0.1% triton-X100. After blocked with 3% BSA for 1 hour the cells were stained with primary (1:50 dilution) and FITC-conjugated secondary antibodies respectively for 1 hour at RT. The positively stained population was detected using a FACSCalibur flow cytometer with 488-nm blue laser and standard FITC 530/30 nm bandpass filter.

### Western blotting analysis

The protein expression levels were determined by staining with primary antibodies and relevant HRP conjugated secondary (1:5000, Armersham, Buckinghamshire, UK) antibodies. The primary antibodies (Santa Cruz, CA, USA: Bcl2, Bax, p65, IκBα, nucleolin, ALDH1A1, 1A3, 3A1, 2 and cleaved PARP; Novus Bio, Cambridge, UK: HIF2; Abcam, Cambridge, UK: phosphorylated p65_S276; Cell Signaling, Herts, UK: AKT, phosphorylated AKT, Sox2, Oct4, JNK, phosphorylated JNK, cJun, phosphorylated cJun, phosphorylated p38, ERK) were diluted at 1:1000 in 5% fat-free milk-TBST. Anti-α-tubulin (1:8000, Sigma) and nucleolin were used as a loading control. The signal was detected using an ECL Western blotting detection kit (GeneFlow, Staffordshire, UK).

### Electrophoretic mobility-shift assays (EMSA)

Nuclear protein extraction was carried out as previously described [21]. A double-stranded NFκB DNA probe (5′-AGT TGA GGG GAC TTT CCC AGG C-3′) was end labeled with biotin. Detection of NFκB-oligonucleotide complex was performed using a LightShift chemiluminescent EMSA kit (Pierce, Northumberland, UK). Briefly, nuclear protein (5 μg) was incubated with 20 fmol of biotin-labeled oligonucleotide for 20 min at room temperature in binding buffer consisting of 10 mM Tris at pH 7.5, 50 mM KCl, 1 mM DTT, 2.5% glycerol, 5 mM MgCl_2_, 50 ng of poly(dIdC), and 0.05% Nonidet P-40. The specificity of the NFκB DNA binding was determined in competition reactions in which a 200-fold molar excess of unlabeled wild type or mutant NFkB probe (5′-AGT TGA TAT TAC TTT TAT AGG C-3′) was added to the binding reaction. Products of binding reactions were resolved by electrophoresis on a 6% polyacrylamide gel. NFκB-oligonucleotide complex was electroblotted onto a nylon membrane (Amersham). After incubation in blocking buffer for 1 hour at room temperature, the membrane was incubated with streptavidin-HRP conjugate for 30 min at room temperature. The membrane was incubated with chemiluminescent substrate for 5 min and exposed to radiographic film.

### Luciferase reporter gene assay

The effect of different treatments on the transcriptional activity of NFκB was determined by luciferase reporter gene assays. Transfections were performed using Lipofectamine 2000 transfection reagent according to the manufacturer's instructions (Invitrogen, Paisley, UK). Cells (1 × 10^4^/well) were cultured in 96-well plates overnight. The luciferase reporter vectors (0.8μg/well) [pNFκB-Tal-Luc (BD Biosciences) and pGL3-Basic (Promega, Southampton, UK)] were co-transfected with 0.008 μg/well pSV40-Renilla (Promega) DNA, an internal control for normalization of the transcriptional activity of the reporter vectors. Twenty-four hours after transfection, the cells were lysed and luciferase activity was determined using Dual Luciferase Assay kit (Promega) according to the manufacturer's instructions. The luciferase activity in each well was normalized to pSV40-Renilla using Ln = L/R (Ln: normalized luciferase activity; L: luciferase activity reading; R: Renilla activity reading). The transcriptional specificity was monitored by the transcriptional activity of the pGL3-Basic. All transfections were performed in triplicate with at least duplicate independent experiments.

### Measurement of ROS activity

The intracellular ROS levels were determined using a Total ROS Detection Kit (Enzo, Exeter, UK) following the supplier's instruction. Briefly, the cancer cells (2×10^5^ cells/well) were cultured in 6-well plate until 70% confluence. The cells were exposed to Lipo-DS (1 μM) in combination with Cu (10 μM). Cells exposed to NAC (2 mM) were used as a negative control. After drug exposure, the drug containing medium was discarded and the cells were collected by trypsinization. The cell pellets were resuspended and incubated in 500 μl of ROS Detection Solution at 37°C for 30 min in the dark. The ROS positive population was detected using a FACSCalibur flow cytometer with 488-nm blue laser and standard FITC 530/30 nm bandpass filter.

### Clonogenic Assay

Cells (5×10^4^/well) were cultured in 6-well plates overnight and then exposed to 1 μM of DS in combination with 10μM CuCl_2_ for 1 h. The cells were collected and cultured in 6-well plates containing drug-free medium at a cell density of 2.5×10^3^/well. Clonogenic cells were determined as those able to form a colony consisting of at least 50 cells after 10 days culture.

### Total RNA isolation and RT-PCR

Total RNA was isolated using TRIzol reagent (Invitrogen, Paisley, UK) according to the manufacturer's protocols. The mRNA expression levels of ALDH 1A1 (NM_000689), 1A3 (NM_000693), 2 (NM_000690) and 3A1 (NM_000691) genes were determined using the Access RT-PCR System (Promega, Southampton, UK) following the instruction of the supplier. The human housekeeping gene GAPDH (XR018317) was used as the RNA loading control. The sequences of the primers and the sizes of the amplified fragments are as follows. 1A1 (154 bp): F: 5'-TGTTAGCTGATGCCGACTTG-3', R: 5'-TTCTTAGCCCGCTCAACACT-3'; 1A3 (150 bp): F: 5'-TCTCGACAAAGCCCTGAAGT-3', R: 5'-TATTCGGCCAAAGCGTATTC-3'; ALDH2 (193 bp) F: 5'- AACCAGCAGCCCGAGGTCTT-3', R: 5'- AAGGTGAGCCCAGCTGGAAG-3'; 3A1 (229 bp) F: 5'-TGTTCTCCAGCAACGACAAG-3', R: 5'-CTGACCTTCAGGCCTTCATC-3'; GAPDH (226bp) F: 5′-GAAGGTGAAGGTCGGAGTC-3′, R: 5′- GAAGATGGTGATGGGATTTC-3′. The RT-PCR conditions were as follows: One cycle at 48°C for 45 min; 1 cycle at 94°C for 2 min; 35 cycles at 94°C for 30 sec, 60°C for 30 sec, 72°C for 1 min; 1 cycle at 72°C for 5 min.

### Human breast cancer xenograft experiments

Five-week-old female BALB/c Nu/Nu athymic nude mice (Biotechology & Cell Biology Shanghai, China) were housed under pathogen-free conditions according to the animal care guidelines of Fourth Military Medical University (FMMU), China. The animal experiments were reviewed and approved by the Ethical Committee of FMMU. The animal study regimen is detailed in Fig. 6. The tumor volume was calculated by the following formula: V =(L×W^2^)×0.5, where L is the length and W is the width of the tumor. The xenograft size was observed twice per week for 4 weeks. After 4 weeks or when the xenograft reached 1500mm^3^, the animals were sacrificed. The tumors were removed, photographed and subjected to further analysis.

### Immunohistochemistry and H&E staining

The paraffin embedded tumor and normal tissue sections were stained using Ki67 (1:200, Cell signaling) and ALDH1 (1:100, Abcam) antibodies then stained with biotinylated anti-mouse immunoglobulin G (H + L) secondary antibody followed by incubation in ABC reagent (DAKO Labs, Cambridgeshire, UK). For H&E staining, the slides were deparaffinized, rehydrated and stained with hematoxylin and eosin for 1 minute. The slide was mounted with 3,3'-diaminobenzidine and visualized under a light microscope.

### Terminal deoxyribonucleotidyl transferase–mediated dUTP nick end labeling (TUNEL) assay

Tumor tissues were paraffin embedded and stained according to the instruction of the manufacturer (Roche, West Sussex, UK). Briefly, the slides were incubated with TdT Enzyme, Stop/Wash Buffer, antidigoxigenenin, and then stained with peroxidase substrate and incubated in ABC reagent. Finally, the slide was mounted with 3,3'-diaminobenzidine and visualized under a light microscope.

### Statistical Analysis

SPSS 13.0 Student's *t* test and one way analysis of variance (ANOVA) followed by Tukey's Multiple Comparison Test were used to calculate the differences. Data were expressed as mean±SD. P≤0.05 was considered as significantly change.
